# Explaining the Use Behavior of Digital Technologies in Pediatric Rehabilitation: Structural Equation Modeling Analysis of a Cross-Sectional European Survey

**DOI:** 10.2196/80355

**Published:** 2026-06-22

**Authors:** Johanne Mensah Gourmel, Sylvain Brochard, Saranda Bekteshi, Elegast Monbaliu, Gwenaël Cornec, Anca Irina Grigoriu, Christopher J Newman, Marco Konings, Javier De La Cruz, Christelle Pons

**Affiliations:** 1 UMR1101, Université de Bretagne Occidentale, Brest, France; 2 Physical Medicine and Rehabilitation Department, Centre Hospitalier Universitaire de Brest, 2 avenue Foch, Brest, 29200, France, 33 292223333; 3 Pediatric Rehabilitation Department, Fondation Ildys, Brest, France; 4 INSERM - UMR1101, Laboratoire de Traitement de l'Information Médicale, Brest, France; 5 Department of Rehabilitation Sciences, Neurorehabilitation Technology Lab, KU Leuven, Bruges, Belgium; 6 Multifactorial Gait Analysis Laboratory, National Center of Neurorehabilitation for Children "Robanescu-Padure", Bucharest, Romania; 7 Paediatric Neurology and Neurorehabilitation Unit, University Hospital of Lausanne, Lausanne, Switzerland; 8 Clinical Sciences Section, Faculty of Biology and Medicine, Lausanne University, Lausanne, Switzerland; 9 UiCEC+12, University Hospital 12 de Octubre Research Institute (imas12), Madrid, Spain

**Keywords:** digital technologies, rehabilitation, pediatrics, Unified Theory of Acceptance and Use of Technology model, UTAUT, structural equation modeling

## Abstract

**Background:**

Digital technologies for rehabilitation (DT4R), such as robotics and treadmill systems (RobTS), virtual reality and active video gaming (VR-AVG), and telehealth and apps (T&Apps), are promising tools for pediatric motor rehabilitation. Identifying acceptance factors is essential for effective clinical adoption.

**Objective:**

This study aimed to analyze the use of 3 different technologies for rehabilitation—RobTS, VR-AVG, and T&Apps—through a causal model based on the Unified Theory of Acceptance and Use of Technology (UTAUT).

**Methods:**

This study was part of RehaTech4child, a cross-sectional survey (2022) supported by the European Academy of Childhood-onset Disability, aimed at professionals working in pediatric motor rehabilitation across Europe. It assessed DT4R use, intention to use, and UTAUT concepts (performance expectancy, effort expectancy, social influence, and barriers). Structural equation modeling was performed to analyze the data and understand relationships between observed and latent variables.

**Results:**

A total of 1397 responses were received, and 635 fulfilled the eligibility criteria. The fitness indices suggested a satisfactory fit between the data and the model. The model explained 67% of the variance in the use of RobTS, 62% in VR-AVG, and 57% in T&Apps. Among all studied determinants, access had the strongest impact on use for all 3 categories of DT4R (RobTS: β=0.78, VR-AVG: β=0.73, and T&Apps: β=0.70; *P*<.001). Intention to use significantly impacted use behavior for all technologies; it was the second determinant after access for VR-AVG (β=0.18, *P*<.001) and T&Apps (β=0.21, *P*<.001), with a lower weight for RobTS (β=0.06, P=.007; *P*<.001). In the subgroup analysis of respondents reporting easy access, intention to use was the strongest determinant of use. The model explained 61% of the variance in intention to use for RobTS, 67% for VR-AVG, and 68% for T&Apps. Performance expectancy had the strongest effect on intention to use for the 3 technologies (RobTS: β=0.81, VR-AVG: β=0.84, and T&Apps: β=0.90; *P*<.001). For this concept, the items with the highest weights were significantly related to the effectiveness of DT4R on rehabilitation. Social influence and effort expectancy had a slight impact on intention to use.

**Conclusions:**

These results underscore the need to ensure easy access as a prerequisite for assessing relevant determinants of acceptance. Developing the evidence base for DT4R effectiveness and ensuring the availability of existing evidence may facilitate DT4R implementation. In our study, within the framework of the UTAUT model, no acceptance barrier was linked to the use of DT4R with children. Gathering families’ views may be useful for the implementation of RobTS. T&Apps may be useful for involving parents in their child’s rehabilitation. Further studies should focus on children’s and families’ points of view.

## Introduction

Motor impairment hinders a child’s development and participation [1,2]. Specialized care and continuous management with individualized rehabilitation programs reduce the burden of disabilities. Keeping rehabilitation fun and engaging to optimize its effect is a real challenge to maintain during growth; including recent technological advances that enable new approaches could help address this issue. Digital technologies (DT), including robotics and treadmill systems (RobTS), virtual reality and active video gaming (VR-AVG), and telehealth and apps (T&Apps), are creating new opportunities in pediatric rehabilitation. Their use shows promise in areas such as upper limb rehabilitation [3], improving walking and balance [4,5], and facilitating the activities and participation of children with disabilities [6,7]. In addition, these technologies can be used to motivate children and encourage their participation [8,9]. Preliminary evidence showed that digital technologies for rehabilitation (DT4R) are both acceptable [10] and enjoyable for children [9]. These 3 groups of technologies represent growing opportunities in pediatric motor care; however, there is still room for optimization regarding their integration into clinical practice. Children who require motor rehabilitation are a heterogeneous population. Furthermore, they grow and their developmental abilities change constantly, which creates specific challenges for the implementation of technologies [11] and supports the need to conduct specific studies. Further research is required to understand the behavioral factors influencing acceptance to improve implementation. In this context, RehaTech4child, a cross-sectional survey (2022) supported by the European Academy of Childhood-onset Disability (EACD), was disseminated to professionals working in pediatric motor rehabilitation across Europe. The first aim was to assess access to and use of DT for pediatric motor rehabilitation in Europe as a function of individual and environmental factors, as well as the potential barriers to their use. The initial results highlighted that DT4R was regularly used in clinical practice by around two-thirds of the European respondents.

The gap between the provision of equipment in a clinical facility and its actual use has been highlighted in the literature [12], and this gap is particularly pronounced for technologies. In the late 1980s [13], theories were developed to explain an individual’s behavior toward a new technology and to understand the levers that may help providers move from purchase to effective practical implementation within their departments. To increase usage, the core concepts highlight the need to improve technology acceptance [12]. In 2003, Venkatesh et al [14] identified 8 models that explain how and why individuals adopt new information technologies and synthesized them into a unique model. Finally, they retained the core concepts that explained the largest proportion of variance in the Unified Theory of Acceptance and Use of Technology (UTAUT) model: approximately 50% of the variance in use and 70% in intention to use. In this model, intention to use and facilitating conditions are the 2 direct determinants of use. Three main determinants are directly related to intention to use: performance expectancy (PE), effort expectancy (EE), and social influence (SI). PE can be defined as the degree to which an individual believes that using the system will help them attain gains and enhance their job performance, EE is defined as the degree of ease associated with the use of the system, and SI is defined as the degree to which an individual perceives that important others believe they should use the system. Facilitating conditions pertain to the existing organizational and technical infrastructure supporting the system use. Applying this conceptual framework to a given technology improves understanding of its acceptance. This can help individuals and teams who purchase and implement technologies within clinical facilities to identify actions aimed at facilitating successful implementation [14].

The UTAUT model has been widely used to explain health care professionals’ behavior toward clinical information systems (eg, the picture archiving and communication system and health information system) and to identify levers and actions to be taken to facilitate their implementation and adoption [15-17]. More recently, the model has also been applied to specific rehabilitation technologies, such as the use of 3D printing systems by occupational therapists [18] and the use of an exergame-based telerehabilitation system by older adults and their health care professionals [19]. Previous UTAUT studies have generally shown that intention to use is an important determinant of use, confirming the need to address behavioral aspects of technology adoption. Regarding the determinants of intention to use, PE is generally a key factor [20], and this effect appears to hold across various professions [21,22]. However, to our knowledge, these studies have remained focused on isolated technologies and contexts, without drawing generalizable insights into the acceptance of rehabilitation technologies. Recent studies [23] highlighted that there are still challenges in the implementation of DT4R, including using these devices to their full potential. The question of the relevance of DT4R for children was also raised in terms of device size and developmental issues [24], as well as cost-effectiveness and patient preferences [25]; however, research in pediatric populations has been focused on technical rather than behavioral aspects. To our knowledge, no study has evaluated the acceptance of digital technologies in pediatric rehabilitation. Children requiring motor rehabilitation have specific needs that must be understood. Furthermore, they are widely dispersed geographically. Therefore, we chose a macroscopic perspective to identify levers and actions to facilitate DT4R implementation in children. The objective was to gather the opinions of a large and diverse European sample across 3 groups of digital technologies to obtain generalizable and actionable findings. Considering technologies with varied levels of technicity and usability by nonprofessionals may help deepen the understanding of acceptance across the different categories.

This study aimed to assess the behavioral components of use and intention to use for RobTS, VR-AVG, and T&Apps in pediatric motor rehabilitation through a causal model based on the UTAUT, and thus to identify key acceptance factors for improving implementation in clinical practice, complementing previous descriptions of access and use in relation to individual and environmental determinants [26].

Based on responses from health care professionals participating in the European RehaTech4Child survey, we hypothesized that (1) the UTAUT model would explain use behavior for the 3 DT4R technologies; (2) intention to use would have the strongest impact on use for each technology; (3) PE would significantly impact intention to use, regardless of profession; and (4) the impact of determinants on use would differ across technologies.

## Methods

### Study Design

This study was part of RehaTech4child, an online European cross-sectional survey (2022) sponsored by the EACD, targeting professionals working in pediatric motor rehabilitation across Europe. This report follows the STROBE (Strengthening the Reporting of Observational Studies in Epidemiology) and CHERRIES (Checklist for Reporting Results of Internet E-Surveys) guidelines. The study was registered on ClinicalTrials.gov (NCT05176522).

### Ethical Considerations

The study was conducted in compliance with current French legislation (Loi Jardé 2012‐300) [27-29]. The Brest CHU (Centre Hospitalo-Universitaire/University Hospital Center) Institutional Review Board considered that ethical committee approval was not necessary.

### Survey Description

The first part of the survey focused on individual determinants (respondents’ sociodemographic characteristics) and environmental determinants, including the type of professional practice and the characteristics of respondents’ patients. The second part assessed DT4R use, intention to use, and UTAUT concepts for each of the 3 categories of DT4R: RobTS, VR-AVG, and T&Apps [8,26]. The UTAUT model is recommended in the health care context but must be adapted to a given technology and the specific context of each study [12,14]. On the basis of previous research and discussions with the steering committee during the elaboration of the survey, we oriented the questions to focus on barriers rather than facilitating conditions to align with previous research on professional practice conditions in rehabilitation, where organizational and technical infrastructures appeared as barriers, as well as feedback from the pilot testers [8,30,31]. Furthermore, in rehabilitation, access has been stated as a key priority to facilitate DT4R adoption [32]. However, access is not always considered a barrier per se in the literature and is not often included as such in studies about barriers and enablers [33]. A previous study showed the major impact of access on use [26]. Access was therefore considered separately from other barriers. Use (and frequency of use), intention to use, PE (8 questions), EE and SI (2 questions), access (and ease of access), and barriers were evaluated on a 7-point Likert scale. The survey is available online [34]. A detailed description of the survey protocol has been published previously [8].

### Participants

The survey was translated into 20 different languages and disseminated by the EACD national coordinators through European and national professional and personal networks using convenience and snowball sampling. Responses were excluded if respondents did not practice motor rehabilitation with children, were from a non-EACD member country, or did not provide responses about DT4R [8,26].

### Data Analysis

**Figure 1.**
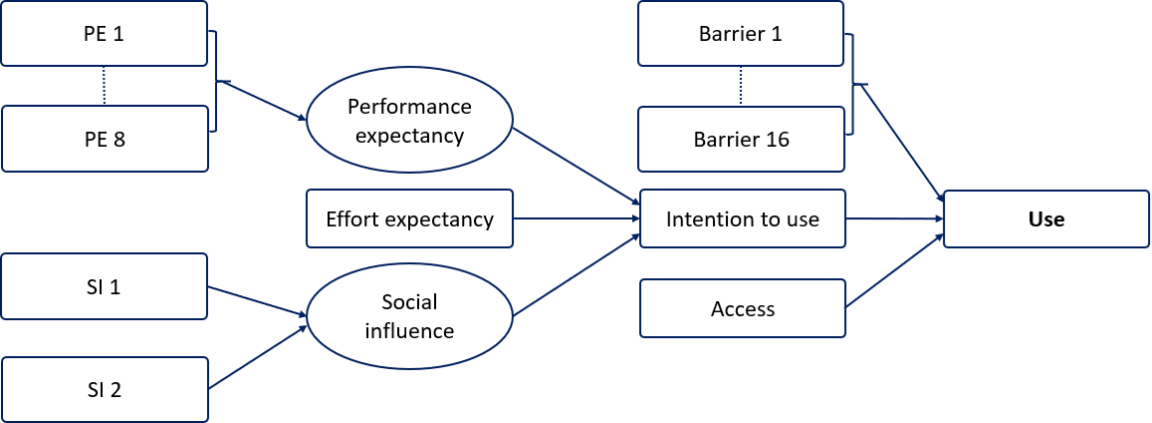
Relationship between observed (rectangular boxes) and latent (oval boxes) variables as specified in the structural equation modeling implemented using R software. The dashed line includes all items in between. SI: social influence; PE: performance expectancy.

Structural equation modeling was used to analyze the data and elucidate the relationships between observed and latent variables, based on the UTAUT model as defined by Venkatesh et al [14] (Figure 1). R software (Lavaan library) was used. Model fit indices were calculated (comparative fit index [CFI], Tucker-Lewis index [TLI], and root mean square error of approximation [RMSEA]) to evaluate the quality of the model, as recommended by Stone [35]. *R*^2^ coefficients were determined for the explained variance of use and intention to use for the 3 DT4R categories in the main analysis. Relationships between latent and observed variables were evaluated using the standardized regression coefficient (β) and its associated *P* value. Statistical significance was set at *P*<.05.

### Subgroup Analysis

As access and ease of access were previously identified as major determinants of use and frequency of use [26], exploratory analyses were performed for subgroups based on ease of access to explore if the impact of various determinants differed for respondents reporting easier access (defined as ≥4 points on the Likert scale).

The literature suggests that EE may be underestimated by individuals who have not experienced the studied technology at least once [12,18]. Therefore, to determine whether this could be a leverage point for improvement to facilitate the implementation of DT4R, we conducted an exploratory analysis of the data for the subgroup of actual users.

Other authors highlighted differences in the impact of SI when studying 2 countries with different cultures [36]. We therefore performed a subgroup analysis on areas of Europe to explore the impact of SI across geographical subregions. The countries listed by Pons et al [8] in the protocol were classified according to the United Nations Geoschemes [37].

Evidence in the literature shows that PE influences the intention to use, and this effect appears to hold across various professions. Therefore, to evaluate this in the specific context of pediatric rehabilitation, we performed a subgroup analysis on professions.

## Results

### Participants’ Characteristics

A total of 1395 responses were received between January 1, 2022, and November 1, 2022, of which 635 were included in the analysis; 599 (94%) responses were complete for RobTS, 563 (89%) for VR-AVG, and 553 (87%) for T&Apps. Of the respondents, 83% (528/635) were female participants. There were 53% (334/635) physiotherapists, 26% (166/635) physicians, and 15% (95/635) occupational therapists. It has been reported that 37% (219/599), 45.5% (256/563), and 50.8% (281/553) of respondents used RobTS, VR-AVG, and T&Apps, respectively. When asked about their intention to use DT4R if they had access to it, 76.6% (459/599), 74.2% (418/563), and 69% (382/553) of all respondents at least somewhat agreed that they would use RobTS, VR-AVG, and T&Apps, respectively. A detailed description of participant characteristics has been published previously [26].

### Structural Equation Modeling: Model Fit Indices

Standard model fit indices showed that the model was acceptable (CFI=0.839/0.871/0.876, TLI=0.818/0.855/0.86, and RMSEA=0.081/.073/0.07 for RobTS, VR-AVG, and T&Apps, respectively).

### UTAUT Determinants of Use

**Figure 2.**
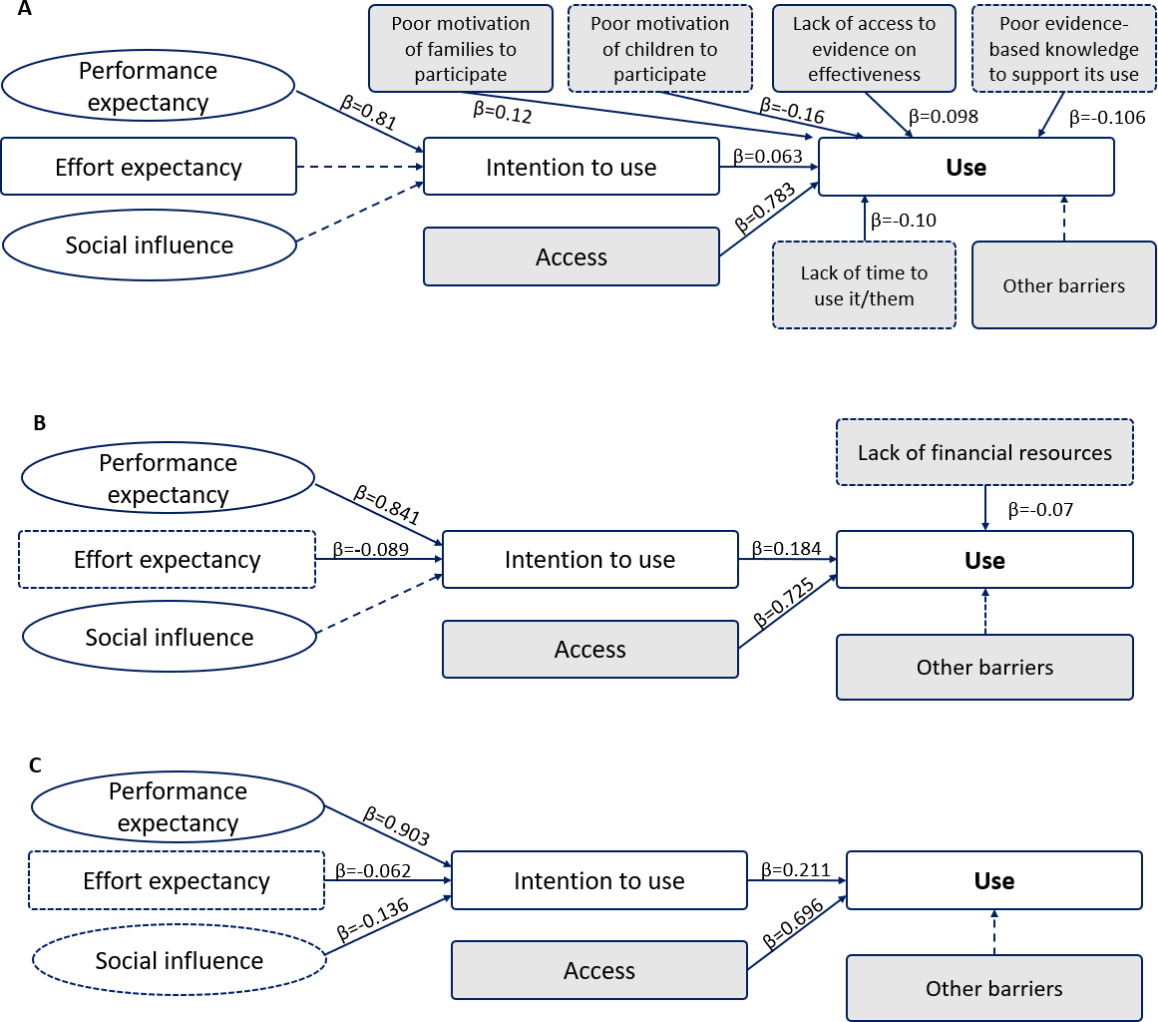
Determinants of use and intention to use for robotics and treadmill systems (A), virtual reality and active video gaming (B), and telehealth and apps (C) according to the Unified Theory of Acceptance and Use of Technology model. Dotted arrows represent β coefficients with *P*>.05. β coefficients are specified when *P*<.05. Dashed box contours indicate negative coefficients. Barriers are represented by light gray boxes. β coefficients were estimated using structural equation modeling. Comparative fit index=0.839/0.871/0.876, Tucker-Lewis index=0.818/0.855/0.86, and root mean square error of approximation=0.081/0.073/0.07 for robotics and treadmill systems, virtual reality and active video gaming, and telehealth and apps, respectively.

The model explained 67% of the variance in use for RobTS, 62% for VR-AVG, and 56% for T&Apps. Among all the variables, access had the strongest impact on use for all 3 categories of DT4R (RobTS: β=0.78, VR-AVG: β=0.73, and T&Apps: β=0.70; *P*<.001) (Figure 2A, B, and C and Table 1).

**Table 1.** Results of the structural equation modeling for use of robotics and treadmill systems (RobTS), virtual reality and active video gaming (VR-AVG), and telehealth and apps (T&Apps) with the standardized regression coefficients (β) and their associated *P* value for each of the latent and observed variables.

Use	RobTS		VR-AVG		T&Apps	
	β	*P* value	β	*P* value	β	*P* value
Intention to use	0.06^a^	.01	0.18^a^	<.001	0.21^a^	<.001
Access	0.78^a^	<.001	0.73^a^	<.001	0.70^a^	<.001
Barrier 1: Lack of financial resources	0.02	.39	−0.07^a^	.02	−0.04	.21
Barrier 2: Lack of confidence (technophobia)	0.01	.84	0.01	.79	−0.02	.63
Barrier 3: Lack of time to learn how to use it/them	0.05	.22	0.01	.91	−0.02	.76
Barrier 4: Lack of time to use it/them	−0.010^a^	.03	−0.07	.17	0.04	.46
Barrier 5: Lack of time to set up and clean up	−0.002	.10	0.04	.42	0.05	.27
Barrier 6: Lack of robustness (in situations like heavy usage, pulling, and drooling)	0.05	.11	0.03	.49	−0.06	.13
Barrier 7: Lack of access to evidence on effectiveness	0.10^a^	.02	−0.002	.97	0.03	.54
Barrier 8: Poor evidence-based knowledge to support its effectiveness and use	−0.12^a^	.01	0.01	.89	−0.03	.63
Barrier 9: Lack of accessible assistance (specialized instruction or specific person)	−0.005	.88	0.003	.94	−0.02	.75
Barrier 10: Lack of initial training	−0.05	.23	−0.08	.15	0.02	.77
Barrier 11: Lack of educational opportunities	0.01	.30	0.04	.58	−0.01	.90
Barrier 12: Lack of training opportunities	−0.10	.05	−0.02	.77	−0.09	.24
Barrier 13: Space-related issues (eg, insufficient or inappropriate space)	0.03	.35	0.001	.97	0.06	.12
Barrier 14: DT^b^ that are not adapted to clients	0.05	.08	−0.01	.82	0.02	.66
Barrier 15: Poor motivation of children to participate	−0.16^a^	<.001	−0.001	.99	−0.07	.19
Barrier 16: Poor motivation of families to participate	0.12^a^	.01	0.05	.26	0.07	.24

a *P*<.05.

b DT: digital technologies.

Intention to use significantly impacted use behavior for all technologies; it was the second determinant after access for VR-AVG (β=0.18) and T&Apps (β=0.21), with a very low weight for RobTS (β=0.06; *P*<.001).

In the subgroup analysis of respondents reporting easier access, among all studied variables, the impact of intention to use was the strongest (RobTS: β=0.41, *P*<.001; VR-AVG: β=0.53, *P*<.001; and T&Apps: β=0.35, *P*<.001). The impact of access was then nonsignificant and slight.

Regarding the other determinants (Table 1), some were statistically significant but had slight weights. For RobTS, barrier 7: “*Lack of access to evidence on effectiveness*” (β=0.098) and barrier 16: *“Poor motivation of families to participate”* (β=0.12) had a statistically significant impact on use, whereas barrier 4: *“Lack of time to use it/them”* (β=–0.10), barrier 8: “*Poor evidence-based knowledge to support its effectiveness and use*” (β=−0.106), and barrier 15: “*Poor motivation of children to participate*” (β=−0.16) had a statistically negative impact on use. For VR-AVG, barrier 1: *“Lack of financial resources”* (β=−0.07) had a statistically negative impact on use.

### UTAUT Determinants of Intention to Use

The model explained 61% of the variance in intention to use for RobTS, 67% for VR-AVG, and 68% for T&Apps. PE had the strongest effect on intention to use for all 3 DT4R categories (β=0.81 for RobTS, β=0.84 for VR-AVG, and β=0.90 for T&Apps; *P*<.001) (Figure 2A, B, and C and Table 2). This effect persisted in the subgroup analysis of professions.

**Table 2.** Results of the structural equation modeling for intention to use of robotics and treadmill systems (RobTS), virtual reality and active video gaming (VR-AVG), and telehealth and apps (T&Apps) with the standardized regression coefficients (β) and their associated *P* value for each of the latent and observed variables.

Structural equation modeling	RobTS		VR-AVG		T&Apps	
	β	*P* value	β	*P* value	β	*P* value
Performance expectancy						
PE^a^ 1: I believe that rehabilitation with DT^b^ complements or enhances the therapists’ abilities	0.87^c^	<.001	0.88^c^	<.001	0.87^c^	<.001
PE 2: I think that the use of rehabilitation DT has the potential to improve motor outcomes	0.89^c^	<.001	0.90^c^	<.001	0.84^c^	<.001
PE 3: I think that the use of rehabilitation DT systems encourages active participation of the patient	0.88^c^	<.001	0.84^c^	<.001	0.83^c^	<.001
PE 4: I think that the use of rehabilitation DT adds value to what a conventional approach offers	0.87^c^	<.001	0.90^c^	<.001	0.88^c^	<.001
PE 5: I think that DT4R^d^ give the opportunity for family members to participate in the rehabilitation process	0.61^c^	<.001	0.64^c^	<.001	0.75^c^	<.001
PE 6: I think that the use of rehabilitation DT is appropriate within my clinical practice	0.72^c^	<.001	0.78^c^	<.001	0.79^c^	<.001
PE 7: I think that the use of rehabilitation DT is a strategy to increase the number of potential clients	0.55^c^	<.001	0.53^c^	<.001	0.58^c^	<.001
PE 8: I think that rehabilitation DT devices make my work more interesting	0.74^c^	<.001	0.78^c^	<.001	0.77^c^	<.001
Social influence						
SI^e^ 1: People whose opinions I value think that I should use rehabilitation DT	0.98^c^	<.001	0.96^c^	<.001	0.99^c^	<.001
SI 2: My supervisor thinks that I should use rehabilitation DT	0.76^c^	<.001	0.78^c^	<.001	0.75^c^	<.001
Intention to use						
PE	0.81^c^	<.001	0.84^c^	<.001	0.90^c^	<.001
EE^f^	−0.05	.06	−0.09^c^	.001	−0.06^c^	.02
SI	−0.046	.20	−0.04	.25	−0.14^c^	<.001

a PE: performance expectancy.

b DT: digital technologies.

c *P*<.05.

d DT4R: digital technology for rehabilitation.

e SI: social influence.

f EE: effort expectancy.

Regarding PE determinants (Table 2), items 1 to 4 evaluated the effectiveness of DT4R on rehabilitation. They had the strongest impact on PE, and their weights were similar. PE 6 evaluated the relevance of DT4R in pediatric rehabilitation, and its impact was slightly lower than that of PE 1 to PE 4. The item PE 5: *“I think that DT4R give the opportunity for family members to participate in the rehabilitation process”* had a moderate impact, apart from T&Apps, for which the impact was higher. PE 7: *“I think that the use of rehabilitation digital technologies is a strategy to increase the number of potential clients”* had the lowest weight for the 3 categories of DT4R.

SI and EE (Table 2) had a slight impact on use in comparison with PE. This effect persisted in the subgroup analysis of European subregions. The impact of the opinions of people whose opinions were valued by the respondents was slightly higher than the impact of supervisors’ opinions.

The exploratory analysis of the results for users found only a slight impact of EE on the intention to use compared with PE.

## Discussion

### Principal Findings

In this study, acceptance was modeled within the framework of the UTAUT model using structural equation modeling for 3 types of DT4R: RobTS, VR-AVG, and T&Apps. The model included data from more than 500 different professionals across Europe working in pediatric motor rehabilitation, with or without experience in pediatric rehabilitation technologies. The model explained 55% to 70% of the variance in intention to use and use, as expected by Venkatesh et al [14]. It also contributed to establishing general principles for rehabilitation technologies while highlighting specific characteristics across the different categories of DT4R. Generalizable insights were first highlighted. Within the UTAUT framework, access had the strongest impact on use among all determinants for all 3 DT4R categories. Furthermore, when access was easier, the impact of intention to use became the strongest. PE was the main determinant of intention to use for all 3 DT4R categories, whereas the impacts of SI and EE were slight. For PE, the items regarding the effectiveness of DT4R on rehabilitation had the highest impact. Specific characteristics were then underlined. For instance, for RobTS, the item on children’s poor motivation was negatively correlated with use, whereas the item on families’ poor motivation was positively correlated. For T&Apps, the item concerning family involvement had a higher weighting than for the other DT4R categories. These analyses helped identify actions needed to promote behavioral change and support the adoption of DT4R.

Lack of access was highlighted as a barrier in the first step of this study [26] and is consistently reported as such in the literature [38]. This barrier is particularly relevant to technologies such as RobTS and VR-AVG, which require space and are relatively expensive. At first sight, the impact of access on use seemed to overwhelm the other determinants of the UTAUT model. However, when access was not only provided but also made easier, intention to use had the strongest impact on use. In summary, ease of access can be understood as a prerequisite for use. Therefore, we recommend conducting a proper evaluation of access conditions before addressing the issue of acceptance for the implementation of DT4R.

PE was the main determinant of intention to use for all 3 DT4R categories. Furthermore, its impact was far greater than that of the other UTAUT determinants. The importance of PE has already been highlighted in the literature on the acceptability of digital technologies in health care, both in the general context of acceptance [12,39,40] and in the specific context of the UTAUT model [41,42] and is now confirmed in the specific context of pediatric rehabilitation. This effect globally persisted in the subgroup analysis of professions. If professionals are convinced that DT4R will improve their practice, they are more likely to use it. This aligns with the items that had higher weights for PE in our study, namely those concerning the effectiveness of DT4R on rehabilitation. To increase intention to use, professionals must first be confident in the performance of DT4R.

To this aim, some of our results could provide an initial lead; however, they carry a slight weight compared to the main determinants. Notably, 2 barriers that had a statistically significant influence on intention to use for RobTS were related to scientific evidence: negatively for the proposition *“poor evidence-based knowledge to support its use”* and positively for *“lack of access to evidence on effectiveness.”* In previous studies, *“lack of evidence for the use of RobTS”* has been highlighted as a limitation to DT4R use by professionals [23], despite the fact that evidence suggests promising results for RobTS in training specific functions, at least in some indications [4]. Our findings suggested that the professionals who responded may already be convinced of the utility of RobTS but were hindered in its use by limited access to evidence. This interpretation should be taken with caution, given the slight weights; however, this information may pave the way for further actions to be taken by rehabilitation department leaders to facilitate the implementation of DT4R in clinical practice: highlighting the evidence of effectiveness (by conducting research in their departments, providing precise recommendations, and facilitating access to current evidence) of DT4R to rehabilitation professionals. Furthermore, digital technology developers should be made aware of this information and should consider developing evidence in accordance with health care guidelines.

The impact of EE on intention to use was lower than that of PE for all 3 DT4R categories. Slegers et al [18] suggested that initial testing of the system reduced perceived ease of use, and Holden and Karsh [12] suggested that EE might be underestimated by people with no or little experience of the technology. To explore this point, we conducted an exploratory analysis of the data for the subgroup of actual users. Although the results of this subgroup analysis should be interpreted with caution, given the smaller sample size, the impact of EE did not change. However, as suggested in previous studies [12], a double impact of experience may occur: having no actual experience of the DT may lead to an underestimation of EE, and, on the contrary, with time and practice, the EE of the system becomes no longer important to acceptance and use. Thus, if the initial training is not sufficient, the EE may increase. However, with sufficient training, it will no longer be a determining factor. In further studies, as done by Nicora et al [23], specifying the level of experience, both in terms of years of practice and practice frequency, may facilitate the analysis of the impact of EE to confirm these hypotheses.

Technical and logistical issues should be considered when implementing DT4R for children, such as screen exposure duration [24], the size of devices, etc. In our acceptance model, the weight of items specifically related to the relevance of DT4R for children, such as “*digital technologies are not adapted to the clients (for professionals working with children*),” was rather slight. Furthermore, the barrier “*poor motivation of children to participate*” was significantly negatively linked to use for RobTS; however, it carried only slight weight. These findings suggest that there was no specific acceptance barrier linked to the use of DT4R in children. Regarding the involvement of families, opinions varied across technologies. Indeed, the barrier “*Poor motivation of families to participate*” was a significant determinant of RobTS use, but with a slight weight. In contrast, evidence in the literature suggests that parents have positive expectations and are generally satisfied with the use of robotic devices by their children [43]. It would be interesting to deepen this specific point in further studies and explore the risk of preconceived ideas of professionals on the motivation of families. On the contrary, some DT4R characteristics could even be assets in the context of pediatric rehabilitation. Thus, in our model, the possibility of involving families more strongly determined PE for T&Apps than for other DT4R categories. For pediatric rehabilitation, T&Apps offer tremendous new possibilities to involve families in their child’s rehabilitation [44]. This specific objective may be considered and valued when developing or purchasing a T&Apps device.

Clinicians directly mediate their patients’ access to DT. Therefore, the factors influencing therapists’ intentions to use the technology are crucial to the implementation process and a condition for access to DT for children and families. To address this, we first questioned health care professionals. However, the overall picture will not be complete without the perspectives of children and families, who are the final users. To ensure efficient use, both the development and evaluation of new digital technologies should involve users. Evaluating acceptance from children’s and families’ perspectives is a priority in future research to avoid a “one-size-fits-all” approach and to ensure personalized and individualized proposals [45]. Interestingly, recent evidence showed that for the deployment of new interventions, including numeric content for children with emotional disorders, parents’ intention to use was influenced by perceived usefulness and efficacy [46]. It would be interesting to determine whether this also applies to DT4R.

SI had a slight impact on the respondents’ intention to use DT4R, in line with previous findings [23], for example, in a study of the use of an exergame-based telerehabilitation system by patients and health care professionals [19]. It has been suggested that SI has a higher impact in mandatory contexts, when the use of a device is imposed [14]. Some studies also found that SI increased in specific contexts, such as the use of diabetic footwear by patients [15]. Moreover, we found studies that reported a high impact of SI; those studies defined SI as the “degree of support for one’s use of technology from individuals or groups that one considers important to oneself” [42], rather than the opinion of significant others on the technology itself.

To our knowledge, few studies based on the UTAUT model have compared the influence of SI through the lens of culture. In another continent, Akiogbe et al [36] showed that SI has a significantly stronger impact on Chinese students than on Japanese students regarding the use of mobile health (mHealth). They noted that in collectivist societies, SI plays a stronger role [36,47] as individuals rely on group norms and institutional endorsements. Metallo et al [48] showed that cultural values may hinder the use of health care technologies due to social norms. The exploratory subgroup analysis found that the impact of SI was not modified by European subregions. However, the subgroup sample was relatively small; therefore, the results should be considered with caution. Further studies could aim to deepen the understanding of the impact of culture on SI.

Furthermore, European countries differ in their institutions, laws, and ethical principles [49], and these differing institutional contexts may affect acceptance.

### Limitations and Future Work

Our model explained approximately 60% to 70% of the variance in intention to use and 55% to 67% of the variance in use, which corresponds to the expected performance of the UTAUT model at its development [12,14] and its current use thereafter. To the best of our knowledge, models that find higher proportions of explained variance are often modified from the original UTAUT model by adding parameters [17,50]. For example, Ngusie et al [17] explained 84.5% of the variance in intention to use with their extended UTAUT3 model, in which they introduced additional constructs such as attitude as a mediator and technology anxiety and digital technology self-efficacy as exogenous variables. This raises the question of the risk of overfitting. Adding parameters may increase the explained variance but limit the generalizability of the results. It also raises the question of the difficulty in respecting the consistency of the UTAUT model, which should be adapted to a given context without being denatured. The UTAUT model was not developed with the aim of explaining all the variance in a use behavior; rather, it was built to be robust. It offers an observational lens, which may be enriched to ensure a comprehensive understanding of professionals’ behavior toward DT4R. That is why we chose to use the UTAUT model without adding additional parameters.

Some fit indices (CFI and TLI) were slightly below commonly cited thresholds. In accordance with recommendations from the methodological literature, these indices were interpreted as guidelines rather than strict decision thresholds. The results were consistent with the literature in terms of the weights of major determinants.

Because of the convenience and snowball sampling methods used to obtain the largest possible sample of European respondents, the sample may not be representative. However, to obtain a representative sample, data on the epidemiological characteristics of rehabilitation professionals would have been necessary; to our knowledge, such data are not available at the European level. We aimed to limit this impact by translating the survey into numerous European languages and disseminating it through national coordinators, national and local professionals, and personal networks. This strategy resulted in responses from 30 out of 43 countries. Nonetheless, the respondents may have had a greater interest in DT4R than the general population of rehabilitation professionals, which could affect the behavioral components of acceptance, especially the intention to use. By disseminating the survey in 20 languages through personal and professional networks, we aimed to reach as varied a sample as possible. Responses were obtained from people with different levels of exposure to and experience with a given technology, but the years of experience with the technology were not gathered in our survey. Further studies could include more precise characterization of exposure levels and experience and propose appropriate strategies according to levels of training and experience to facilitate implementation, as done by Nicora et al [23]. In that Italian study, respondents were stratified according to their years of experience in robot-assisted rehabilitation.

Some studies that combined the perspectives of professionals and patients on a given technology have shown that their views may differ. Seinsche et al [19] showed that health care professionals underestimated the ability of patients to properly set up and use the studied exergame-based telerehabilitation system. Therefore, further studies aiming to evaluate the opinions of children and their relatives would be enlightening, especially given that the 3 DT4R categories, like those studied by Seinsche et al [19], and in contrast with digital technologies such as imaging or shared medical record software, are likely to be directly used by patients and families, especially in the context of remote rehabilitation and telehealth.

Some of the standardized coefficients, although statistically significant, were small. The results should be explored further by additional studies.

We conducted subgroup analyses to improve understanding of the impact of effort expectancy, ease of access, European subregions, and professions. However, the subgroup samples were small; therefore, the results should be interpreted with caution.

### Conclusions

This study modeled acceptance based on the UTAUT framework for 3 categories of DT4R—RobTS, VR-AVG, and T&Apps—in pediatric motor rehabilitation. Data were gathered from more than 500 respondents across Europe, and responses were compared across the 3 categories. We then proposed actions to improve acceptance and, consequently, the implementation of DT4R. Within the framework of the UTAUT model, access and the conditions required for easy access emerged as prerequisites for evaluating DT4R acceptance, regardless of the type of DT4R. It is known that offering access to a technology is not sufficient to ensure its implementation within a clinical facility. However, rehabilitation centers must provide and assess access conditions before assessing behavioral barriers and facilitators. Acceptance of DT4R was positively influenced by PE, the main determinant of intention to use for all 3 DT4R categories, outweighing the other determinants. These findings suggest that, regardless of the type of DT4R being implemented in a clinical setting, improving acceptance and bridging the gap between access and effective use require efforts to demonstrate and communicate the effectiveness of the device. To achieve this aim, the elaboration of evidence-based clinical guidelines explicating indications and protocols for use (including, for instance, session length and frequency) is warranted. DT development would benefit from being grounded in the scientific evidence of what works and what does not. The weights of SI and EE on intention to use were slight. In our study, within the framework of the UTAUT model, no acceptance barrier was linked to the use of DT4R in children. Gathering families’ views may be useful for the implementation of RobTS. T&Apps may be assets for involving families in their child’s rehabilitation. Limitations included self-reported measures and the use of convenience and snowball sampling methods. A large and diverse European sample was chosen to identify actionable and generalizable levers; however, this should be subsequently adapted to local contexts. Further studies should focus on children’s and families’ points of view.

## Abbreviations

CFI: comparative fit index
CHERRIES: Checklist for Reporting Results of Internet E-Surveys
DT4R: digital technologies for rehabilitation
EACD: European Academy of Childhood-onset Disability
EE: effort expectancy
mHealth: mobile health
PE: performance expectancy
RMSEA: root mean square error of approximation
RobTS: robotics and treadmill systems
SI: social influence
STROBE: Strengthening the Reporting of Observational Studies in Epidemiology
T&Apps: telehealth and apps
TLI: Tucker-Lewis index
UTAUT: Unified Theory of Acceptance and Use of Technology
VR-AVG: virtual reality and active video gaming
